# Optimization of Sperm Cryopreservation Formulation in *Portunus trituberculatus*

**DOI:** 10.3390/ijms24054358

**Published:** 2023-02-22

**Authors:** Le Chang, Chengpeng Lu, Junquan Zhu, Yiner Chen, Chunlin Wang, Changkao Mu, Congcong Hou

**Affiliations:** Key Laboratory of Applied Marine Biotechnology by the Ministry of Education and Key Laboratory of Marine Biotechnology of Zhejiang Province, School of Marine Sciences, Ningbo University, Ningbo 315822, China

**Keywords:** *Portunus trituberculatus*, sperm cryopreservation, sperm survival rate, cryopreservation formulation

## Abstract

*Portunus trituberculatus* is a very important marine economic species, and its aquaculture industry has been developing rapidly. However, the phenomenon of marine wild capture of *P. trituberculatus* and germplasm degradation has become increasingly serious. It is necessary to develop the artificial farming industry and carry out germplasm resource protection, for which sperm cryopreservation technology is an effective method. This research compared three methods (mesh-rubbing, trypsin digestion, and mechanical grinding) for acquiring free sperm, and the best method was mesh-rubbing. Then, the optimal cryopreservation conditions were selected, and the optimal formulation was sterile calcium-free artificial seawater, the optimal cryoprotectant was 20% glycerol, and the best equilibrium time was 15 min at 4 °C. The optimal cooling program was suspending the straws at 3.5 cm on the liquid nitrogen surface for 5 min and then storing them in liquid nitrogen. Finally, the sperm were thawed at 42 °C. However, the expression of sperm-related genes and the total enzymatic activities of frozen sperm were significantly decreased (*p* < 0.05), which showed that sperm cryopreservation damaged the sperm. Our study improves the sperm cryopreservation technology and the yield of aquaculture in *P. trituberculatus.* Additionally, the study provides a certain technical basis for the establishment of a sperm cryopreservation library of crustaceans.

## 1. Introduction

*Portunus trituberculatus* belongs to Arthropoda, Crustacea, Decapoda, Portunidae, and *Portunus*, which is a famous large marine economic crab [1]. They have high nutritional value and medicinal value [2]. The main distribution of *P. trituberculatus* is in Japan, Korea, the Malay Archipelago, and many coastal areas in China [3,4]. However, with the rapid development of the marine aquaculture industry, the aquaculture environment is deteriorating dramatically, natural germplasm resources are severely degraded, and disease outbreaks are frequent, which not only cause huge economic losses but also strongly restrict the sustainable and healthy development of the aquatic animal husbandry industry [5,6]. Therefore, the conservation of germplasm resources has been a major problem to be solved urgently in the aquaculture industry of *P. trituberculatus*, and the most mature solution to this problem is the cryopreservation technology of sperm and egg [7].

Sperm cryopreservation can preserve sperm for a long time, maintain some excellent traits of sperm, and can avoid various problems for in vivo conservation due to gene loss, generation interval, and genetic drift [8,9]. Second, it can greatly save the cost of breeding and provide sperm or embryos for scientific research or production anytime and anywhere without the limitations of time and space, which can protect genetic diversity to a certain extent [10]. Third, cryopreservation technology can minimize the interference of external environmental factors on experimental animal germplasm resources and prevent the spread of animal-derived diseases. In addition, cryopreservation technology can provide high-quality materials for artificial breeding and cultivating new varieties, thus constantly expanding the breeding of a good trait population [11,12]. Without cryopreservation preservation, good breeds can only be bred for several generations. Finally, cryopreservation technology has opened up a new way of establishing a germplasm resource bank [13].

Cryopreservation has applications in animal husbandry, aquaculture, biotechnology, and the conservation of threatened species [14,15]. Cryopreservation of mammalian sperm and embryos has brought great convenience to the livestock industry; for example, frozen semen of cattle has been commercialized and can be routinely used in breeding programs [16,17,18]. In aquatic animals, to break through geographical isolation and developmental asynchrony, researchers have conducted studies on the cryopreservation of sperm for some aquatic economic species. More than 200 species of fish semen have been successfully preserved [19,20], which is of great value to the conservation of fish germplasm resources and the study of fish genetic breeding. Additionally, corresponding cryopreservation studies have been conducted on the sperm of aquatic species such as crustaceans [13,21,22], echinoderms [23,24], and mollusks [25,26]. This is increasingly important to the germplasm conservation of aquatic animals. However, research on the cryopreservation of crustacean sperm is relatively underdeveloped, unsystematic, and insufficient and significantly lags behind that of vertebrates. Additionally, there are no studies on the cryopreservation of *P. trituberculatus* sperm.

The cryopreservation process mainly includes sperm collection and quality evaluation, preparation of diluent, selection and preparation of cryoprotectants, freezing, liquid nitrogen storage, thawing, and evaluation of sperm quality after thawing [23,27]. In particular, the types and concentrations of cryoprotectants, diluents, cooling procedures and thawing procedures will have a great impact on sperm quality [13]. Due to the great differences in sperm’s physiological characteristics between different types of crabs, there are no universal diluents and cryoprotectants at present [13,21,22]. In different studies, researchers have selected diluents and cryoprotectants based on information from the literature combined with their own experience. Currently, studies on the cryopreservation of *P. trituberculatus* sperm are lacking. The purpose of this study was to select the optimal cryopreservation method for *P. trituberculatus* by screening various sperm-acquisition methods and cryoprotectant combinations and to provide a scientific basis for the reproductive regulation and breeding of *P. trituberculatus*. This study is important for improving the germplasm, selecting and breeding excellent species, and improving the aquaculture production of *P. trituberculatus*. It has wide research significance and application prospects in the conservation of species diversity.

## 2. Results

### 2.1. Sperm Collection

More than 90% free sperm of *P. trituberculatus* were obtained by 0.2% trypsin digestion and underwent an acrosome reaction; the sperm survival rate was 8.2%. More than 60% of the free sperm obtained by the mesh-rubbing method were normal, and the sperm survival rate was 64.3%. However, the sperm obtained using the mechanical grinding method was seriously damaged, and the sperm survival rate was 10.7%. Almost all sperm died after freezing.

### 2.2. The Morphological Evaluation of Sperm

**Figure 1:**
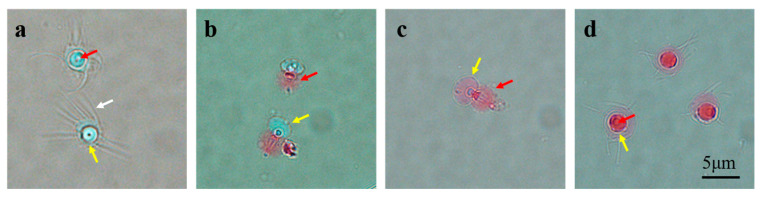
Observation of live and dead sperm under a light microscope at ×1000 magnification. (**a**) Live sperm. (**b**) Live sperm with acrosome reaction. (**c**) Dead sperm with acrosome reaction. (**d**) Dead sperm. Red arrow: acrosome. Yellow arrow: nucleus. White arrow: radical arm.

We observed that the type Ⅰ live sperm did not undergo an acrosome reaction and was not colored after eosin B staining. The spherical sperm lacks flagellum but has radical arms, and their main functional internal organizations are an acrosome (inner circle in Figure 1a) and a nucleus (external circle in Figure 1a). The acrosome of type II sperm was inversed, and only the acrosome part was colored red (Figure 1b). The acrosome of type III sperm was inverted, and the whole sperm was colored red (Figure 1c). The acrosome morphology of type Ⅳ sperm remained normal, but the whole sperm was colored red (Figure 1d).

### 2.3. Cryopreservation of Sperm in P. trituberculatus

#### 2.3.1. Effects of Different Kinds and Concentrations of Cryoprotectants

**Figure 2:**
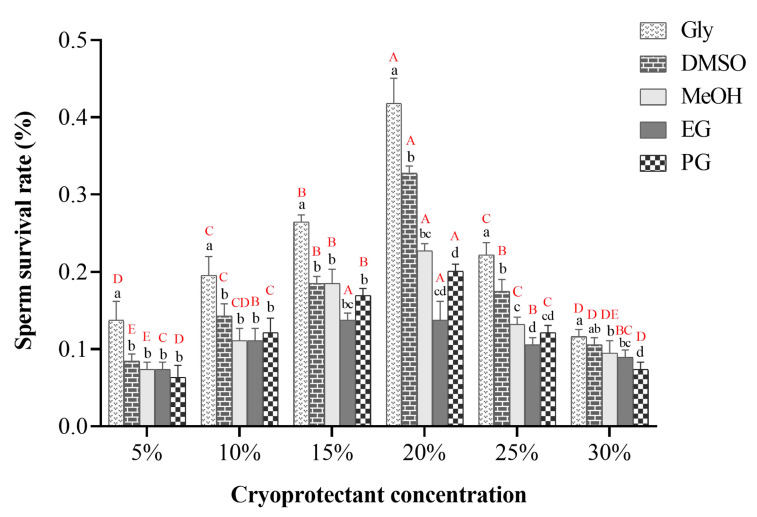
Survival rates of sperm of *P. trituberculatus* after the application of different kinds and concentrations of cryoprotectants. Different lower-case letters indicate the significant difference in the freezing effect of different cryoprotectants at the same concentration (*p* < 0.05). Different capital letters indicate differences in the freezing effect of different concentrations of the same cryoprotectant (*p* < 0.05). Gly: glycerol, DMSO: dimethyl sulfoxide, MeOH: methanol, EG: ethylene glycol, PG: propylene glycol (*n* = 60).

The sperm survival rate was significantly different among different types and concentrations of cryoprotectants for sperm of *P. trituberculatus*. Glycerol was better than the other cryoprotectants in each of the six different concentration groups. Among them, 20% glycerol was the most effective cryoprotectant, with a sperm survival rate of 41.79%, which was significantly better than other concentrations of glycerol (*p* < 0.05) (Figure 2). The worst was 5% propylene glycol, with a sperm survival rate of only 6.35% (Figure 2). In conclusion, glycerol was the most suitable cryoprotectant for cryopreservation of sperm in *P. trituberculatus*, and DMSO was the second most effective.

#### 2.3.2. Effects of Different Equilibrium Times at 4 °C

**Figure 3:**
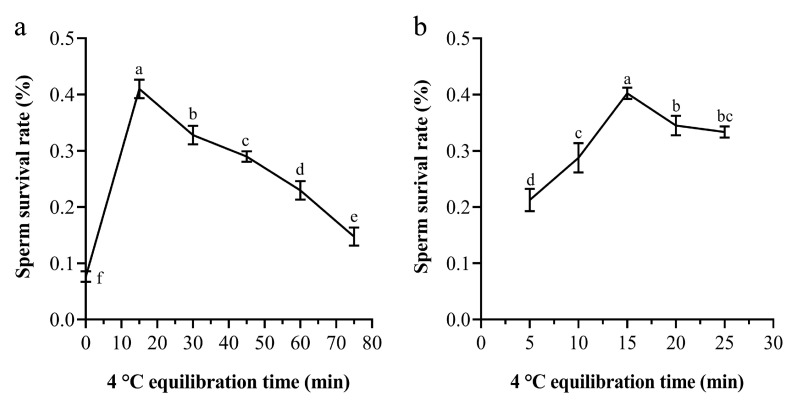
Survival rates of sperm of *P. trituberculatus* after different equilibration times. (**a**) Survival rates of sperm after 0, 10, 20, 30, 40, 50, 60, 70, and 80 min. (**b**) The survival rate of sperm after 5, 10, 15, 20, and 25 min. The same letter means that the difference is not significant (*p* > 0.05), and different letters mean that the difference is significant (*p* < 0.05) (*n* = 60).

The survival rates of frozen sperm of *P. trituberculatus* after cryopreservation at different equilibrium times were significantly different. The results in Figure 3a show that the freezing effect at 4 °C for 15 min was the best, which was significantly higher than that in the other groups (*p* < 0.05), and the sperm survival rate was 40.98%. The experimental group without balanced direct input of liquid nitrogen had the worst effect, and the sperm survival rate was only 7.65% (Figure 3a). After narrowing the time gradient, 15 min was still the best equilibrium time when other cryopreservation conditions remain unchanged (Figure 3b). More or less than 15 min will reduce the survival rate of sperm, and the longer the time was, and the more or less time than 15 min, the lower the survival rate of sperm.

#### 2.3.3. Effects of Different Cooling Procedures on Cryopreservation

**Figure 4:**
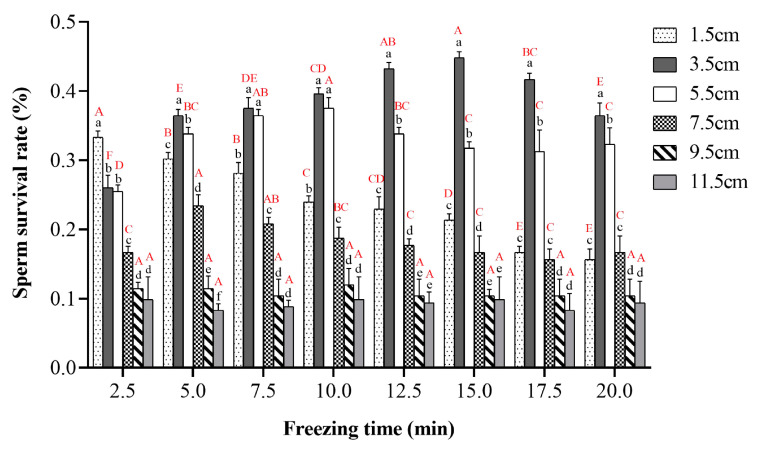
Survival rates of sperm of *P. trituberculatus* after different cooling procedures on cryopreservation. Different lower-case letters indicate significant differences between straws at different heights (*p* < 0.05). Different capital letters represent significant differences between different times at the same height (*p* < 0.05) (*n* = 60).

The results showed that the most suitable cooling height was 3.5 cm above the liquid nitrogen surface for the horizontal position of the straw. The best effect was staying for 15 min at 3.5 cm above the liquid nitrogen surface, where the sperm survival rate was 44.79% (Figure 4). The sperm survival rate was 43.22% when the straw was kept at 3.5 cm on the liquid nitrogen surface for 12.5 min, which was not significantly different from the former (*p* > 0.05). The sperm survival rate was 36.46% when the straw was kept at 3.5 cm on the liquid nitrogen surface after 5 min, which was significantly different from that at 3.5 cm for 15 min (*p* < 0.05) (Figure 4).

#### 2.3.4. Effects of Different Thawing Temperatures

**Figure 5:**
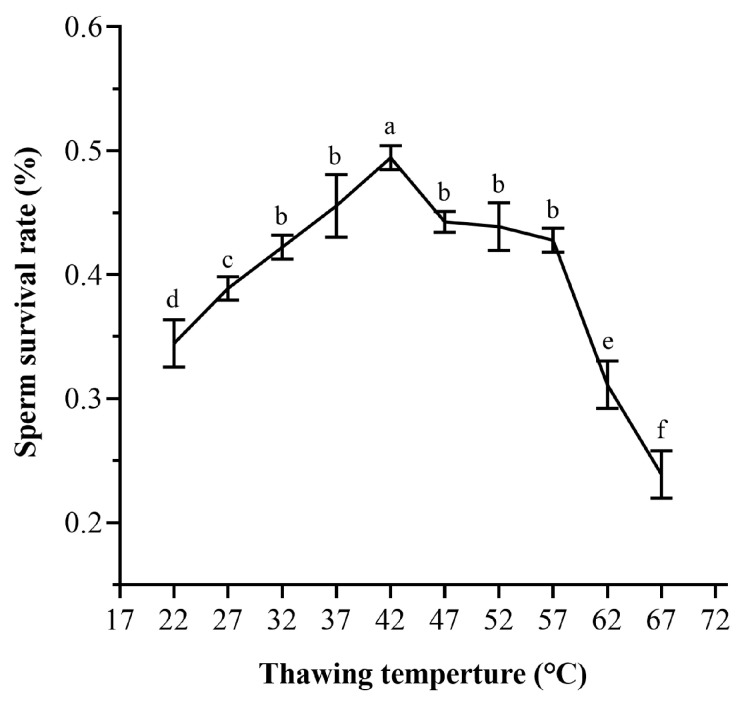
The survival rates of sperm of *P. trituberculatus* after different resuscitation temperatures on cryopreservation. The same letter means that the difference is not significant (*p* > 0.05), and different letters mean that the difference is significant (*p* < 0.05) (*n* = 60).

As shown in Figure 5, under different thawing temperatures, the best survival rate of frozen sperm was the water bath at 42 °C, where the sperm survival rate can reach 49.44%, followed by the water bath at 37 °C, where the survival rate of sperm was 45.56%. There was no significant difference in the effect of the water bath at 47, 52, and 57 °C (*p* > 0.05), which were 44.26, 43.89, and 42.78%, respectively (Figure 5). The worst effect was thawing at 67 °C, where the survival rate was only 23.89% (*p* < 0.05) (Figure 5).

#### 2.3.5. Effects of Different Freezing Times on Cryopreservation

**Figure 6:**
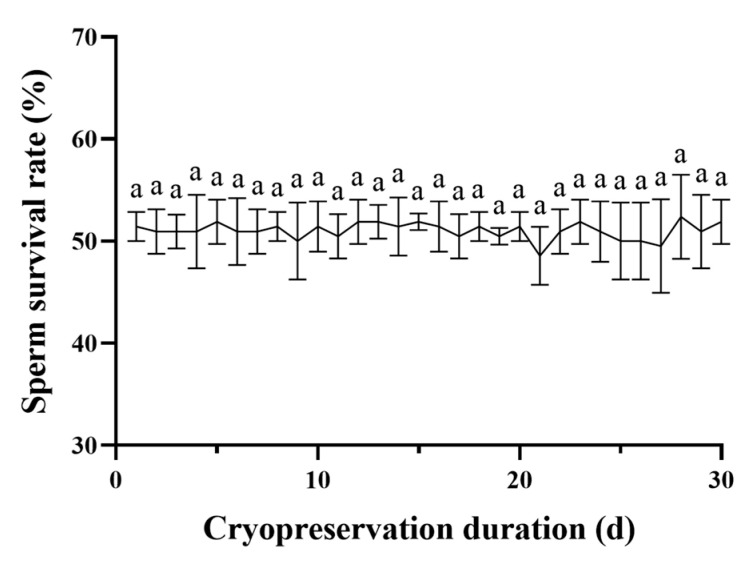
Survival rates of *P. trituderculatus* sperm after different freezing times. There were no significant differences between the survival rate of 1–30 d cryopreservation sperm. The same letter means that the difference is not significant (*p* > 0.05) (*n* = 60).

Under the same other freezing conditions, the survival rate of sperm stored at cryopreservation was detected within 1–30 days, and the sperm survival rate was basically stable at 50%, without a significant difference (*p* > 0.05) (Figure 6).

### 2.4. Gene Expression and Enzyme Activity Analysis of Fresh and Frozen Sperm

#### 2.4.1. Sperm-Related Gene Expression of Fresh and Frozen Sperm

**Figure 7:**
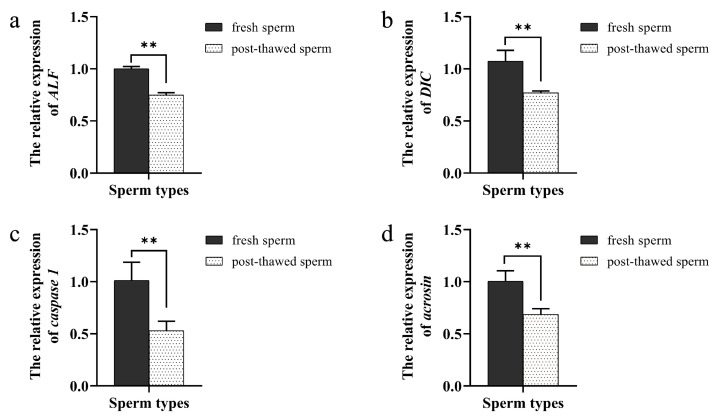
The expression of sperm-related gene in *P. trituberculatus* fresh and frozen sperms. (**a**) The expression of *Aalpha/beta-like factor* (*ALF*). (**b**) The expression of *DIC*. (**c**) The expression of *caspase 1*. (**d**) The expression of *acrosin* and ** means that the difference is significant (*p* < 0.05) (*n* = 60).

The qPCR results showed that the expression of the sperm-related genes *ALF*, *DIC*, *caspase 1* and *acrosin* in frozen sperm was significantly decreased (*p* < 0.05) (Figure 7). ALF is a spermatogenesis-related gene, and its abnormal expression contributes to our molecular understanding of male infertility [28]. *Caspase 1* indicates the sperm’s ability to protect themselves [29]. *DIC* is an important subunit of dynein, and it plays an important role in spermatogenesis in *P. trituberculatus* [30,31]. Acrosin, located in the acrosome of crustaceans, plays an important role in the sperm acrosome reaction [32]. Therefore, *ALF*, *DIC*, *caspase 1*, and *acrosin* can be markers to react sperm viability.

#### 2.4.2. Enzyme Activity Analysis of Fresh and Frozen Sperm

**Figure 8:**
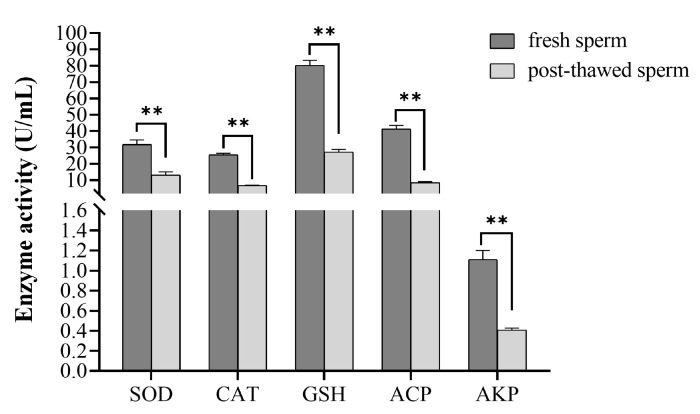
The enzyme activity analysis of fresh and frozen sperms. ** means that the difference is significant (*p* < 0.05) (*n* = 60).

Compared with fresh sperm of *P. trituberculatus*, the enzyme activities of total SOD, catalase (CAT), reduced glutathione (GSH), acid phosphatase (ACP), and alkaline phosphatase (AKP) in frozen sperm were significantly decreased (*p* < 0.05) (Figure 8). The activities of total SOD, CAT, GSH, ACP, and AKP in fresh sperm were 31.81 ± 2.82, 25.51 ± 0.81, 80.21 ± 3.09, and 41.32 ± 2.07 U/mL, respectively, and the activities of total SOD, CAT, GSH, ACP, and AKP in frozen sperm were 13.13 ± 1.89, 6.60 ± 0.23, 27.10 ± 1.63, 8.41 ± 0.66, and 0.41 ± 0.20 U/mL, respectively.

## 3. Discussion

### 3.1. Comparison of Different Methods of Acquiring Free Sperm of P. trituberculatus

The reproductive system of crustaceans is special. Sperms mature in the male reproductive system in the form of spermatophores [33]. Post-mating female crabs also have a large number of free sperm in the spermatheca and constantly exist in dense blocky form. Different techniques are used to collect spermatophores of crustaceans, including electrical extrusion and simple pressure [34,35,36]. If we need to obtain free sperm, we must destroy the spermatophores, which is bound to cause damage to the sperm [37,38]. Currently, the common methods for obtaining free sperm include the mesh-rubbing method, the trypsin digestion method, and the mechanical grinding method. The mechanical grinding method is simple and has been used in sperm acquisition of *C. japonica* [39] and *E. sinensis* [40], but the effect was not good. We found that the number of sperm of *P. trituberculatus* obtained by the mechanical grinding method was small, the damage to the sperm was serious, and almost all of the sperm died after cryopreservation. Trypsin is a hydrolase that specifically hydrolyses peptide bonds formed by the amino group of lysine and the hydroxyl group of arginine. The main component of spermatophores in crustaceans is mucopolysaccharide. Trypsin can effectively digest the wall of spermatophores to obtain free sperm [41]. In this study, we referred to the optimal trypsin digestion conditions for acquiring sperm of *E. sinensis* [42] and found that more than 90% of the sperm had acrosome reactions, which may be due to the significant difference in the size of sperm between *P. trituberculatus* and *E. sinensis*. The sperm of *P. trituberculatus* are relatively small, which is not suitable for 0.2% trypsin. Compared with the other two methods, the mesh-rubbing method is simple, the damage to sperm was minimal, and the sperm deformity rate was low. We found that more than 60% of the total sperm obtained by the mesh-rubbing method were normal. This is consistent with the view that the mesh-rubbing method is the best method for acquiring sperm of *L. vannamei* [43].

The decapod sperm lacks a true flagellum and is immobile, so to date, sperm motility has not been considered to determine sperm quality in decapods [44]. At present, sperm motility evaluation methods used in decapods include morphological observation [45], osmotic pressure method [13], sperm acrosome reaction [46], biological staining [47], and so on. In this study, we referred to the method of Guan et al., and we used the eosin B staining method to evaluate the sperm mortality of *P. trituberculatus* [48]. It is a relative biological indicator that is mainly based on the metabolism of organisms, thus quickly discharging the pigment into the extracellular space.

### 3.2. The Most Suitable Cryopreservation Conditions of Sperm in P. trituberculatus

#### 3.2.1. The Most Suitable Cryoprotectants

In this study, sperm cryopreservation solution was mainly composed of a basic diluent and a cryoprotectant. The diluent can provide a suitable physiological environment and certain nutrition for sperm. It can also reduce the toxic effect of cryoprotectants on sperm and make sperm more vital [20]. Therefore, the diluent should be nontoxic or minimally toxic, and the composition should be as simple as possible. Currently, there is no uniform standard for sperm cryopreservation diluents of mammals, birds, fish, shellfish, and crabs, but there are some references for the diluents between similar species. Studies have shown that artificial seawater has been widely used in sperm cryopreservation in crustaceans [13,46]. In this experiment, because Ca^2+^ can cause sperm aggregation and decrease sperm viability [49], we used sterile Ca^2+^-free artificial seawater as the diluent for sperm cryopreservation, which could ensure the osmotic pressure requirements of the sperm and avoid spontaneous acrosome reactions of sperm [50].

Cryoprotectants can freely penetrate the cell membrane, reduce cell permeability damage during freezing and thawing, and prevent the formation of ice crystals to protect sperm [49]. Currently, the commonly used cryoprotectants are DMSO, glycerol, methanol, EG, PG, and so on [20]. Glycerol, as one of the natural components in lipid metabolism, is the earliest cryoprotectant used. Glycerol can quickly enter the sperm and accelerate the process of dehydration, thereby reducing the formation of ice crystals in sperm [51]. DMSO has also been widely used in cryopreservation of sperm in marine fish and invertebrates, which is related to its good cryoprotective effect on sperm and suitability for most species [19]. The optimal concentration range of DMSO is 5–20%. A high concentration of DMSO easily penetrated the cells, and the cytotoxicity was very obvious. DMSO at the same concentration caused greater damage to sperm than glycerol. In addition, different concentrations of EG, PG, and methanol were also used for the study of sperm cryopreservation of invertebrates, but their effects were not as good as those of DMSO and glycerol. Therefore, a reasonable selected concentration and types of cryoprotectants are crucial for the cryopreservation of sperm. Our study found that 20% glycerol was the optimal cryoprotectant for *P. trituberculatus* sperm (Figure 2). This is consistent with the findings of Bhavanishankar, who suggested that sperm in glycerol have a higher survival rate than those in DMSO [13]. However, the optimal glycerol concentration of *Scylla serrata* sperm was 12.5%, which may be determined by species specificity. Anchordoguy et al. considered 5% DMSO to be the most suitable cryoprotectant for sperm of *Sicyonia ingentis* [46]. Liu et al. suggested that 5% DMSO is the best cryoprotectant for *C. japonica* [35]. Ke and Cai considered the cryoprotectant composed of 10% DMSO and 5–10% glycerol was to be most effective cryoprotectant for cryopreservation of *P. chinensis* sperm, and the sperm survival rate was more than 60%, which was better than a single cryoprotectant [51]. This indicated that different animal sperm had different adaptability and sensitivity to cryoprotectants, and sometimes the combined use of cryoprotectants would produce better results.

#### 3.2.2. The Most Suitable Equilibrium Times at 4 °C

The mixture of cryoprotectant and semen after a certain period of time is conducive to the role of cryoprotectant. With the decrease in temperature, the metabolic activity of sperm is also reduced. The sperm can gradually adapt to the low-temperature environment with a 4 °C balance and will not make the sperm suddenly enter the ultralow temperature environment and cause sperm death. However, different scholars hold different views on how long sperm need to be balanced at 4 °C before cryopreservation. Liao et al. thought it is necessary to balance *M. nipponense* shrimp sperm at 4 °C for 30 min [52]. Xu et al. believed that the effect of balancing the sperm of *Charybdis japonica* at 4 °C for 30 min was better [33]. Ke and Cai believed that it was necessary for the sperm of *P. chinensis* to balance at 0–4 °C for 20–30 min before cryopreservation, and sperm would not undergo an acrosome reaction [51]. Chen believed that the sperm of *E. sinensis* should be balanced at 4 °C for more than 30 min after adding cryoprotectant. If the balance time was less than 30 min, the DNA damage to sperm was obvious. If the sperm were put directly into liquid nitrogen under imbalance, the sperm could not survive after thawing [34]. Our results showed that the time of sperm balance before cryopreservation is different, and the effect is also different. From 0 to 75 min, with the decrease in balance time, the sperm survival rate increased. However, when the equilibrium time was less than 15 min, the sperm survival rate decreased with increasing time (Figure 3). The optimal balance time of *P. trituberculatus* sperm is 15 min, and too long or too short will reduce the survival rate of sperm. Therefore, a balance time at 4 °C had a significant effect on the quality of sperm cryopreservation of *P. trituberculatus*. Therefore, in the process of sperm cryopreservation, it was necessary to balance for 15 min to avoid the damage caused by insufficient balance time.

#### 3.2.3. The Most Suitable Cooling Procedures and Thawing Temperatures

The cooling rate will directly affect the freezing effect of sperm. If the cooling rate is too fast or too slow, it will cause sperm damage. The step-by-step cooling method is more conducive to the adaptation of sperm to the environment to maintain a complete structure [53]. In our study, we used the straw-freezing method. A total of 0.25 mL of the straw containing the sperm of *P. trituberculatus* in a liquid nitrogen surface 3.5 cm was incubated for 15 min and then placed in liquid nitrogen, and the effect of cryopreservation was best (Figure 4). This was different from the results of Zhouˊs previous studies. Zhou believes that the best effect is to put the sperm of *P. trituberculatus* in 0.5 mL straw, drop it slowly in liquid nitrogen vapor (within 10 min), and finally put the straw into liquid nitrogen [54]. This may be due to the continuous solidification of liquid in the process of ultra-low-temperature freezing to form the amorphous and highly viscous state of glass crystallization. The main characteristics of vitrification were a high concentration of cryoprotectants and instantaneous cooling. However, the range of 0–40 °C brings a risk of irreversible mechanical damage to cells, and appropriate cooling options enable cells to move across this range [55] so that they can be put into liquid nitrogen for long-term preservation. Therefore, two-step or three-step cooling methods are generally used in ultra-low-temperature cryopreservation of sperm.

Whether the resuscitation method is appropriate or not affects the cryopreservation effect of sperm to a large extent, different resuscitation methods should be selected for different cooling processes. The effect of resuscitation speed is related to the type of cells, the protective liquid used in cryopreservation, the cooling speed, and the temperature control. Common resuscitation methods are 30–40 °C water-bath thawing and room-temperature water thawing. The thawing effect of *Penaeus chinensis* sperm at 35–40 °C is the best [51]. The sperm survival rate of *Penaeus monodon* was highest after thawing in a 30 °C water bath [36]. The sperm survival rate of *L. vannamei* was the highest after thawing in a 37 °C water bath [56]. The most suitable thawing temperature of *Scylla serrata* sperm was 55 °C [11]. In the thawing process of E. sinensis sperm, Chen used the same temperature as for *Scylla serrata* and achieved good results [34]. In this study, we found that there was no significant difference in thawing *P. trituberculatus* sperm in a water bath at 32–57 °C (except 42 °C) (*p* > 0.05), and the survival rate of sperm was the highest after thawing in a 42 °C water bath (Figure 5). This was close to the thawing temperature selected by most previous studies. This suggests that water bath thawing was suitable for sperm recovery in most animals.

### 3.3. Sperm Cryopreservation Damages the Sperm of P. trituberculatus

Sperm cryopreservation can generate cellular damage that compromises the integrity of the plasma membrane, the viability, and the fertilization capacity of sperm [57]. mRNA molecules are crucial molecules that could be directly related to sperm quality. Methods based on qPCR analysis were recently developed for the detection and quantification of sperm mRNA transcripts in fish, which are important in fertilization and offspring development [58,59]. Cryopreservation induces a decrease in the levels of certain RNAs in sperm and is a useful biomarker of post-thaw quality [58]. Cryopreservation can alter the chromatin structure in sperm, and this process might also affect mRNA stability by altering their association with certain proteins. In our study, the expression of the sperm-related genes *ALF*, *DIC*, *caspase 1*, and *acrosin* in frozen sperm was significantly decreased (*p* < 0.05) (Figure 7). The results showed that cryopreservation can cause some damage to the sperm of *P. trituberculatus*. ALF regulates spermatogenesis as a testis-specific transcription factor, and the abnormal expression of *ALF* is related to abnormal human spermatogenesis [28]. Additionally, the decreased expression of *ALF* mRNA may indicate that cryopreservation decreases sperm fertilization ability. Caspase 1 plays a pivotal role in the immune defense mechanisms against infections by the innate immune system [29]. In our results, the decreased expression of *caspase 1* mRNA may indicate that cryopreservation impairs sperm’s ability to protect themselves and decreases sperm’s fertilization ability. DIC is an important submit of dynein, and the decrease in *DIC* mRNA expression indicates that the function of dynein would be affected [30,31]. Acrosin, a serine protease present in the sperm acrosome, plays an important role in the fertilization process [32]. In our results, the reduction in *acrosin* mRNA expression may predict a decrease in sperm’s fertilization ability. Therefore, sperm cryopreservation can cause some damage to the sperm of *P. trituberculatus*. Additionally, the decrease in gene expression may be the molecular mechanism of sperm vitality decrease in *P. trituberculatus*.

Spermatogenesis in the testis is composed of a series of biochemical reactions, which are regulated by a variety of enzymes [60]. The activity of related enzymes in the testis reflects the overall physiological activity of the testis, and the enzyme as a catalyst for metabolism in the organism is a necessary factor for tissue cells to exert their physiological functions. Therefore, the activity of the enzyme can be used as a basis for determining the physiological activity of the testis [61]. SOD, CAT, ACP, AKP, and GSH have usually been used as the activity markers of testis [62,63,64,65,66,67,68,69,70,71,72,73]. SOD, CAT, and GSH play an important role in the oxidation and antioxidant balance of the body. They can effectively reduce the formation of reactive oxygen species (ROS), such as hydroxyl radicals (•OH) and hydrogen peroxide (H_2_O_2_) [62,63]. If excessive free radicals could not be cleared by the body in a timely manner, they will attack a variety of biological macromolecules, causing DNA damage, enzyme inactivation and lipid peroxidation, and a series of oxidative damage, leading to sperm damage and decreased vitality. They therefore play a vital role in protecting cells from damage and protecting sperm [64,65]. Additionally, GSH activity was correlated with motility, vitality, the mitochondrial state, the oocyte binding capacity, and fertilization of sperm cells [66]. Liu et al. indicated that the enzyme activity in goat semen had a significant effect on sperm motility. Liu et al. also found a SOD and GSH-PX plays an important antioxidant role in goat sperm and they have significant correlation with sperm viability [67,68]. Our results showed that the activities of SOD, CAT, and GSH in *P. trituberculatus* sperm were significantly decreased after cryopreservation (Figure 8), indicating that sperm cryopreservation resulted in a reduction in antioxidant capacity, so many free radicals could not be cleared by antioxidant enzymes, sperm in *P. trituberculatus* was bound to be damaged by oxidation, and sperm viability was decreased. Some studies have shown that •OH and ROS have a greater damaging effect on cells [69,70]. Therefore, sperm cryopreservation has some degree of damage to the sperm of *P. trituberculatus*. Therefore, the activity of antioxidant enzymes (SOD, CAT, GSH) can reflect the physiological status of sperm to a certain extent and can be used as an important physiological index to measure the preservation effect of cryoprotectants.

ACP and AKP are directly involved in the transfer and metabolism of phosphate groups in the defense mechanism of crustaceans [71], which can accelerate the uptake and transport of substances, form a hydrolytic enzyme system, destroy and eliminate foreign bodies, and achieve the defense function of the body [72,73]. They can directly affect the metabolism and vitality of sperm. In addition, ACP can also participate in the synthesis of related proteins under the induction of sex hormones [61]. Thereby, they can be used as the basis for evaluating sperm viability. In our study, we found that the activities of ACP and AKP in frozen sperm were decreased (Figure 8), indicating that sperm cryopreservation influenced the metabolism and vitality of *P. trituberculatus* sperm. Additionally, sperm cryopreservation damages the sperm of *P. trituberculatus*. In sum, the decrease in enzyme activity (SOD, CAT, ACP, AKP, and GSH) may be the mechanism of sperm vitality decrease in *P. trituberculatus*.

### 3.4. Optimal Sperm Cryopreservation Formulation and Its Application

Based on the results of the study, we obtained the optimal sperm cryopreservation formulation of *P. trituberculatus*, which is shown in Table 1. We used this formulation for the 30 d freezing and thawing experiment and found that sperm viability was good and that the sperm survival rate remained stable at approximately 50% during the experiment (Figure 6). The effect was better than that in the study by Zhou et al., in which the sperm survival rate of *P. trituberculatus* sperm was only 25% after freezing for 236 h [49]. However, the sperm survival rate after cryopreservation in our study was less than the sperm survival rate of Scylla serrata sperm cryopreservation in the study of Guan et al., which was 58.7% [42]. Additionally, the sperm survival rate in our study was lower than the sperm survival rate of *Macrobrachium nipponense* sperm cryopreservation in the study of Liao et al., which was 60% [47]. This may be caused by species differences and different cryopreservation formulations; later, we will use different cryoprotectants in combination to optimize the cryopreservation formulation and further improve the sperm survival rate.

## 4. Materials and Methods

### 4.1. Spermatophore Collection

The *P. trituberculatus* crabs used in our experiments were selected from the ChouPijiang farm of Ningbo Zhejiang, China. Healthy and post-mating female *P. trituberculatus* subjects with similar growth conditions were selected for the experiment, with a mean weight of 200 ± 15 g. The crabs were immersed in 1% potassium permanganate solution for 20 min, and then the spermathecas were isolated with a sterilized dissecting knife and immediately placed in sterile calcium (Ca^2+^)-free artificial seawater. After that, the spermathecas were punctured to squeeze out the spermatophores and then stored at 4 °C for subsequent experiments.

### 4.2. Sperm Collection

#### 4.2.1. Trypsin Digestion Method

The fresh spermatophores were placed on clean filter paper to absorb the water, weighed, and then transferred to centrifuge tubes (Jet Biofil, Guangzhou, China). Then, 3 mL of 0.2% trypsin solution was added, and the samples were placed in a water bath at 37 °C for 5 min (the mixture was reversed twice to fully react). The suspension was centrifuged at 500× *g* for 10 min at 4 °C, and the supernatant was then centrifuged at 2000× *g* for 8 min at 4 °C. The precipitate was washed three times with Ca^2+^-free artificial seawater, and then sterile Ca^2+^-free artificial seawater was added to obtain the pure sperm cells. The sperm survival rate in suspension was detected (*n* = 20).

#### 4.2.2. Mesh-Rubbing Method

The fresh spermatophores were placed on clean filter paper to absorb water, weighed, placed into 250 mesh silk screens, placed into a container with (4 °C) sterile Ca^2+^-free artificial seawater, and gently scrubbed to release sperm. Then, the homogenate was transferred into a centrifuge tube and centrifuged at 500× *g* for 10 min at 4 °C, and the supernatant was then centrifuged at 2000× *g* for 8 min at 4 °C. The precipitate was washed three times with Ca^2+^-free artificial seawater, and then sterile Ca^2+^-free artificial seawater was added to obtain the pure sperm cells. The sperm survival rate in suspension was detected (*n* = 60).

#### 4.2.3. Mechanical Grinding Method

The fresh spermatophores were drained and weighed, placed in (4 °C) sterile Ca^2+^-free artificial seawater. After 10 min of settlement at 4 °C, the supernatant was discarded, and the precipitate of spermatophores was resuspended with the same buffer and then gently ground and triturated using glass homogenizer in an ice bath (4 °C) to create a spermatophore homogenate, which was later settled for a further 10 min. Then, the supernatant was transferred to a centrifuge tube and later centrifuged at 500× *g* for 10 min at 4 °C to separate the spermatozoal suspension and the precipitable fragments of spermatophores. The spermatozoal suspension was then centrifuged at 2000× *g* for 8 min at 4 °C. The spermatozoal pellet was washed three times with Ca^2+^-free artificial seawater, and then sterile Ca^2+^-free artificial seawater was added to obtain the pure sperm cells. The sperm survival rate in suspension was detected (*n* = 20). The percentage of sperm viability between the three collect methods was analyzed using a *t*-test. The level of significance for all analyses was set at *p* < 0.05.

### 4.3. Sperm Viability Assessment

The sperm suspension was diluted to 10^7^ sperm ml^−1^ with the aid of a hemocytometer plate (16 × 25 cells in the counting area, total volume of 0.1 mm^3^). The 2% eosin B (MACKLIN, Shanghai, China) staining solution was prepared with sterile Ca^2+^-free artificial seawater. The sperm solution and eosin B staining solution were added to the carrier glass and mixed, and then the glass coverslip was slowly covered. Microscopic examination (Nikon NI-U light microscope, Nikon Corporation, Tokyo, Japan) of 200 sperm was randomly performed (repeated 3 times), and the sperm malformation rate was determined. The sperm was divided into four categories, including (Ⅰ) live sperm without acrosome reaction, (Ⅱ) live sperm with acrosome reaction, (Ⅲ) dead sperm without acrosome reaction, and (Ⅳ) dead sperm with acrosome reaction. We observed that type Ⅰ sperm without acrosome reactions were not colored. The acrosome of type II sperm was inversed, and only the acrosome part was colored red. The acrosome of type III sperm is inversed, and the whole sperm is colored red. The acrosome morphology of type Ⅳ sperm remained normal, but the whole sperm was colored red. Since live sperm with acrosome reactions are no longer capable of insemination, they are not included in the later viability calculations.

### 4.4. Screening of Cryopreservation Conditions for Sperm of P. trituberculatus

#### 4.4.1. Different Kinds and Concentrations of Cryoprotectants

We selected five cryoprotectants, dimethyl sulfoxide (DMSO), glycerol, methanol, ethylene glycol (EG), and propylene glycol (PG) (Sinopharm Chemical Reagent Co., Ltd., Shanghai, China), based on their extensive use for cryopreservation of fish, shrimp, and crab sperm. Each cryoprotectant solution was prepared to final concentrations of 5, 10, 15, 20, 25, and 30% (*v*/*v*) using sterile Ca^2+^-free artificial seawater as the diluent. The sperm solution and the cryoprotectant solution were mixed at a ratio of 1:1 and equilibrated at 4 °C for 30 min. Then, the sperm were stored in liquid nitrogen for 1 h after the two-step temperature-reduction method (plastic straws were placed horizontally at 3.5 cm on the liquid nitrogen surface of a homemade simple cooling device for 5 min). The sperm were thawed in a water bath at 42 °C, and the sperm survival rate was detected; three replicates were conducted for each treatment. We applied one-way ANOVA to compare differences between the sperm survival rate at different kinds of cryoprotectants or different concentrations of cryoprotectants. Significant differences between treatments were identified using Tukey’s HSD and Tukey s-b post hoc tests for post hoc multiple comparisons.

#### 4.4.2. Different Balancing Times at 4 °C

The sperm solution and the cryoprotectant of 20% glycerol were mixed at a ratio of 1:1, and the samples were aspirated into 0.25 mL straws and equilibrated at 4 °C for 0, 15, 30, 45, 60, or 75 min. Then, the sperm were stored in liquid nitrogen for 1 h after the two-step temperature reduction method (method the same as 4.4.1). The sperm were thawed in a water bath at 42 °C, and the survival rate was detected (repeated 3 times). After the optimal equilibration time was obtained, we reduced the equilibrium time gradient. The samples were aspirated into 0.25 mL straws and equilibrated at 4 °C for 5, 10, 15, 20, or 25 min. The other conditions and operation methods were the same as above, and the sperm survival rate was detected. Three replicates were conducted for each treatment. We applied one-way ANOVA to compare differences between the sperm survival rate at different balancing times at 4 °C. Significant differences between treatments were identified using Tukey’s HSD and Tukey s-b post hoc tests for post hoc multiple comparisons.

#### 4.4.3. Different Heights and Times for the Two-Step Temperature-Reduction Method

The sperm solution and the cryoprotectant of 20% glycerol were mixed at a ratio of 1:1, and the samples were aspirated into 0.25 mL straws and equilibrated at 4 °C for 15 min. After that, the sperm were cooled at the liquid nitrogen surface (1.5, 3.5, 5.5, 7.5, 9.5, or 11.5 cm) for 0, 2.5, 5, 7.5, 10, 12.5, 15, 17.5, or 20 min, respectively. The sperm were stored in liquid nitrogen for 1 h and then thawed in a water bath at 42 °C, and the survival rate of the sperm was detected. Three replicates were conducted for each treatment. We applied one-way ANOVA to compare differences between the sperm survival rate at different heights or times for the two-step temperature-reduction method. Significant differences between treatments were identified using Tukey’s HSD and Tukey s-b post hoc tests for post hoc multiple comparisons.

#### 4.4.4. Different Thawing Temperatures and Freezing Times

The sperm solution and the cryoprotectant of 20% glycerol were mixed at a ratio of 1:1, and the samples were aspirated into 0.25 mL straws and equilibrated at 4 °C for 15 min. Then, the sperm was stored in liquid nitrogen for 1 h after the two-step temperature reduction method (method the same as 4.4.1). The sperm was stored in liquid nitrogen for 1 h and then thawed in a water bath at 22, 27, 32, 37, 42, 47, 52, 57, 62, or 67 °C, and the survival rate of the sperm was detected. Three replicates were conducted for each treatment.

**Figure 9:**
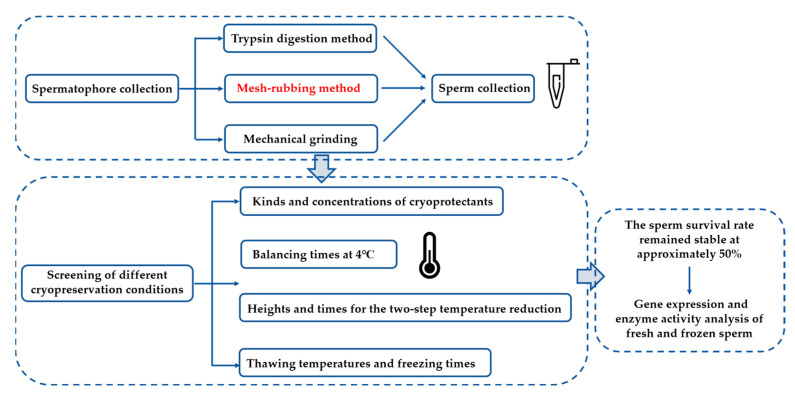
The flow chart of the sperm cryopreservation procedure in *P. trituberculatus*.

The sperm solution and the cryoprotectant of 20% glycerol were mixed at a ratio of 1:1, and the samples were aspirated into 0.25 mL straws and equilibrated at 4 °C for 15 min. Then, the sperm was stored in liquid nitrogen for 1 h after the two-step temperature reduction method (method the same as 4.4.1). The sperm was stored in liquid nitrogen and then thawed in a water bath at 42 °C, and the survival rate of the sperm was detected within 30 days. Three replicates were conducted for each treatment. The flow chart of the sperm cryopreservation procedure was as follows (Figure 9). We applied one-way ANOVA to compare differences between the sperm survival rate at different thawing temperatures or freezing times. Significant differences between treatments were identified using Tukey’s HSD and Tukey s-b post hoc tests for post hoc multiple comparisons.

### 4.5. Gene Expression and Enzyme Activity Analysis of Fresh and Frozen Sperm

#### 4.5.1. Sperm-Related Gene Expression of Sperm in *P. trituberculatus*

Total RNA was extracted from fresh and frozen sperm of *P. trituberculatus* by RNA solv Reagent (OMEGA, Norcross, GA, USA). The PrimeScript™ RT reagent Kit (Takara, Dalian, China) was used for normal reverse transcription. All reverse transcription products (cDNA) were stored at −20 °C. Real-time quantitative PCR (qPCR) was used to analyse the expression of *ALF* (Aalpha/beta-like factor), *caspase 1*, *DIC* (dynein intermediate chain), and *acrosin* in fresh and frozen sperm in *P. trituberculatus.* The primers used for qPCR were designed using Primer Premier 5 software (Premier Biosoft International, Palo Alto, CA, USA) on the cDNA sequences of *ALF*, *caspase 1*, *DIC,* and *acrosin* of *P. trituberculatus* downloaded from the National Center for Biotechnology Information website (https://www.ncbi.nlm.nih.gov/ (accessed on 1 December 2021), NCBI). The GenBank numbers are XM_045276087.1, XM_045248050.1, XM_045252879.1, and OL840042. The primers used for qPCR are listed in Table 2. The *gapdh* gene was used as a positive active control, and the primers were designed based on the *gapdh* cDNA sequence (CenBank Number: EU919707.1).

The Rotor-Gene 6000 System was used for the qPCR assay. A 20 μL reaction volume was set up to carry out the amplification process, containing 5 μL of 1:25 diluted cDNA, 10 μL Master Mix (GenStar, Beijing, China), 1 μL of each primer (10 μM), and 3 μL PCR-grade water. qPCR was carried out at 95 °C for 2 min, followed by 40 amplification cycles (denaturation at 95 °C for 15 s, annealing at 60 °C for 15 s, and extension at 72 °C for 30 s). The comparative delta–delta Ct method was used to analyze the gene expression of *ALF*, *caspase 1*, *DIC*, and *acrosin* in fresh and frozen sperm. The gene expression between the fresh and frozen sperm was analyzed using a t-test. The level of significance for all analyses was set at *p* < 0.05.

#### 4.5.2. Enzyme Activity Analysis of Fresh and Frozen Sperm

The same batch of fresh semen and frozen semen solutions were centrifuged at 4 °C for 8 min at 2000× *g*, and 9 times the volume of physiological saline was added to the suspension. The supernatant was centrifuged at 2500× *g* for 10 min, and the supernatant was obtained. A BCA reagent kit (Beyotime, Shanghai, China) was used to measure the protein concentration.

The glutathione (GSH) content assay kit, catalase assay kit, total SOD activity assay kit, acid phosphatase assay kit, and alkaline phosphatase assay kit (Nanjing Jiancheng Bioengineering Institute, Nanjing, China) were used to detect the enzymatic activities of SOD, CAT, GSH, ACP, and AKP in the same batch of fresh and frozen sperm (repeated three times). All the above operations were carried out according to the instructions provided by the manufacturer. The enzyme activity between the fresh and frozen sperm was analyzed using a t-test. The level of significance for all analyses was set at *p* < 0.05.

### 4.6. Statistical Analysis

The results are presented as the mean ± standard deviation (SD). All analyses and graphs were performed at a significance level of *p* < 0.05 using IBM SPSS Statistics 25.0 (SPSS Software, Chicago, IL, USA) and GraphPad Prism software 8.0.2 (GraphPad Software Inc., San Diego, CA, USA).

## 5. Conclusions

In the process of cryopreservation and thawing, sperm damage mainly includes supercooling shock, ice crystal damage, hypertonic shock, and cryoprotectant toxicity. The current research primarily aimed to improve the survival rate of sperm by adjusting the parameters in the cryopreservation process to reduce the damage caused by these factors. In our study, different sperm acquisition methods and cryoprotectant combinations were screened to select the most suitable method for cryopreservation of *P. trituberculatus* to improve the breeding basis of *P. trituberculatus*, ensuring that sperm supply was not limited by time and space, and to provide basic knowledge for artificial in vitro fertilization. This study has extensive research significance and application prospects in protecting species diversity. It is of great significance to improve the germplasm of *P. trituberculatus*, select and cultivate excellent varieties of *P. trituberculatus*, and improve the yield of aquaculture. At the same time, it provides a certain technical basis for the establishment of the sperm cryopreservation library of crustaceans. In the later stage, different cryoprotectants can be combined to optimize the quality of frozen sperm.

## Figures and Tables

**Table 1:** Optimal sperm cryopreservation formulation of *P. trituberculatus* sperm.

Collection of Sperm	Extender and Cryoprotectants and Dilution Ration	Equilibration Time and Freezing Vessel	Cooling Procedure and Thawing Conditions
Mesh-rubbing method	Sterile Ca^2+^-free artificial seawater; 20% glycerol; 1:1	15 min at 4 °C; 0.25 mL straw	15 min at 3.5 cm above the liquid nitrogen surface, then put into liquid nitrogen; 42 °C water bath

**Table 2:** The primer sequences used in this research.

Primer Name	Sequence (5′ to 3′)	Purpose
ALF-F	ACAACGACTCGGTGGACTTCA	qPCR
ALF-R	TGTGACGAGTCCGTTCTGTAAAG	qPCR
caspase1-F	GGAAGTGGATGCTGGGGGAC	qPCR
caspase1-R	TGTTACACGGTCAAAGTAGCGAT	qPCR
DIC-F	GATGGAGAGGCTAACGAGGC	qPCR
DIC-R	AGGTGATACAGCGATTACGGG	qPCR
acrosin-F	GGCTGGAGTTTGTGAGGCTGG	qPCR
acrosin-R	CTCGTATTGCGGGTGTGGAAT	qPCR
GAPDH-F	GGTTGTGGCGGTGAATGAT	qPCR
GAPDH-R	CTCGGGCTTCATCTCATTGTAT	qPCR

## Data Availability

The authors declare that they have no known competing financial interests or personal relationships that could have appeared to influence the work reported in this paper.

## References

[1] Hamasaki K., Fukunaga K., Kitada S. (2006). Batch fecundity of the swimming crab *Portunus trituberculatus* (Brachyura: Portunidae). Aquaculture.

[2] Zhang X.S., Huang C.K., Guo C., Xie S.C., Luo J.X., Zhu T.T., Yuan Y., Jin M., Zhou Q.C. (2021). Effect of dietary carbohydrate sources on the growth, glucose metabolism and insulin pathway for swimming crab, *Portunus trituberculatus*. Aquac. Rep..

[3] Zhao Y., Kang X., Shang D., Zhai Y., Ning J., Ding H., Sheng X. (2020). Study of Cd Content Distribution and Its Bioaccessibility in Edible Tissues of Crab *Portunus trituberculatus* from the Coastal Area of Shandong, China. Biol. Trace. Elem. Res..

[4] Wu Q., Wang J., Chen R.S., Huang J.X., Zuo T., Luan Q.S., Jin X.S. (2016). Biological characteristics, temporal-spatial distribution of *Portunus trituberculatus* and relationships between its density and impact factors in Laizhou Bay, Bohai Sea, China. J. Appl. Ecol..

[5] Ng’ambi J.W., Li R., Mu C., Song W., Liu L., Wang C. (2016). Dietary administration of saponin stimulates growth of the swimming crab *Portunus trituberculatus* and enhances its resistance against *Vibrio alginolyticus* infection. Fish Shellfish Immunol..

[6] Liu X., Lei Y., Ren Z., Zhou S., Qian D., Yu Y., Yin F., Wang C. (2020). Isolation, characterization and virulence of *Mesanophrys* sp. (Ciliophora: Orchitophryidae) in farmed swimming crab (*Portunus trituberculatus*) in eastern China. J. Fish Dis..

[7] Yoshida M. (2000). Conservation of sperms: Current status and new trends. Anim. Reprod. Sci..

[8] Okutsu T., Yano A., Nagasawa K., Shikina S., Kobayashi T., Takeuchi Y., Yoshizaki G. (2006). Manipulation of fish germ cell: Visualization, cryopreservation and transplantation. J. Reprod. Dev..

[9] Carmichael C., Westerfield M., Varga Z.M. (2009). Cryopreservation and in vitro fertilization at the zebrafish international resource center. Methods Mol. Biol..

[10] Li S., Hu J. (2017). Research progress on cryopreservation of laboratory animal resources. Mod. J. Anim. Husb. Vet. Med..

[11] Cabrita E., Sarasquete C., Martínez-Páramo S., Robles V., Beiro J., Pérez-Cerezales S., Herraez M.P. (2010). Cryopreservation of fish sperm: Applications and perspectives. J. Appl. Ichthyol..

[12] Asturiano J.F., Cabrita E., Horváth Á. (2017). Progress, challenges and perspectives on fish gamete cryopreservation: A mini-review. Gen. Comp. Endocrinol..

[13] Bhavanishankar S., Subramoniam T. (1997). Cryopreservation of spermatozoa of the edible mud crab *Scylla serrata* (Forskal). J. Exp. Zool..

[14] Holt W.V. (1997). Alternative strategies for the long-term preservation of spermatozoa. Reprod. Fertil. Dev..

[15] Anger J.T., Gilbert B.R., Goldstein M. (2003). Cryopreservation of sperm: Indications, methods and results. J. Urol..

[16] Mocé E., Vicente J.S. (2009). Rabbit sperm cryopreservation: A review. Anim. Reprod. Sci..

[17] Sharma R., Kattoor A.J., Ghulmiyyah J., Agarwal A. (2015). Effect of sperm storage and selection techniques on sperm parameters. Syst. Biol. Reprod. Med..

[18] Bhakat M., Mohanty T.K., Raina V.S., Gupta A.K., Khan H.M. (2011). Frozen semen production performance of *Murrah buffalo* bull. Buffalo Bull..

[19] Liu Y., Li X., Robinson N., Qin J. (2015). Sperm cryopreservation in marine mollusk: A review. Aquacult. Int..

[20] Li J.C., Ke W.J., Yang H., Yu H., Yang Y. (2019). Research progress of cryopreservation technology applied to fish sperm preservation. Jiangsu Agric. Sci..

[21] Nimrat S., Siriboonlamom S., Zhang S., Xu Y., Vuthiphandchai V. (2006). Chilled storage of white shrimp (*Litopenaeus vannamei*) spermatophores. Aquaculture.

[22] Kang X., Li G., Mu S., Guo M., Ge S. (2009). Acrosome reaction of Chinese mitten-handed crab *Eriocheir sinensis* (Crustacea: Decapoda) spermatozoa: Promoted by long-term cryopreservation. Aquaculture.

[23] Dunn R.S., McLachlan J. (1973). Cryopreservation of echinoderm sperm. Can. J. Zool..

[24] Asahina E., Takahashi T. (1978). Freezing tolerance in embryos and spermatozoa of the sea urchin. Cryobiology.

[25] Ieropoli S., Masullo P., Santo M., Sansone G. (2004). Effects of extender composition, cooling rate and freezing on the fertilisation viability of spermatozoa of the Pacific oyster (*Crassostrea gigas*). Cryobiology.

[26] Dong Q., Huang C., Eudeline B., Tiersch T.R. (2005). Systematic factor optimization for cryopreservation of shipped sperm samples of diploid pacific oysters, *Crassostrea gigas*. Cryobiology.

[27] Xu S., Sun J.C., Liu S.L., Lin C.G., Zhang L.B., Sun L.N., Yang H.S. (2021). Progress in research on cryopreservation technology for echinoderm sperm. Prog. Fish. Sci..

[28] Huang M., Wang H., Li J., Zhou Z., Du Y., Lin M., Sha J. (2006). Involvement of ALF in human spermatogenesis and male infertility. Int. J. Mol. Med..

[29] Winkler S., Hedrich C.M., Rösen-Wolff A. (2016). Caspase-1 regulates autoinflammation in rheumatic diseases. Z. Rheumatol..

[30] Stuchell-Brereton M.D., Siglin A., Li J., Ahmed S., Williams J.C., Cooper J.A. (2011). Functional interaction between dynein light chain and intermediate chain is required for mitotic spindle positioning. Mol. Biol. Cell.

[31] Wei C.G., Mu D.L., Tang D.J., Zhu J.Q., Hou C.C. (2021). Expression and functional analysis of cytoplasmic dynein during spermatogenesis in *Portunus trituberculatus*. Cell Tissue Res..

[32] Chang L., Xiang Q.M., Zhu J.Q., Chen Y.E., Tang D.J., Zhang C.D., Hou C.C. (2022). Transport of Acrosomal Enzymes by KIFC1 via the Acroframosomal Cytoskeleton during Spermatogenesis in *Macrobrachium rosenbergii* (Crustacea, Decapoda, *Malacostracea*). Animals.

[33] Yang W.X. (1998). A review of organelle changes and functions during spermatogenesis of decapod crustacean. Donghai Marine Science.

[34] Amadio L.M., Mantelatto F.L. (2009). Description of the Male Reproductive System of the Hermit Crab *Calcinus Tibicen* (Decapoda: *Anomura*: Diogenidae). J. Crustacean Biol..

[35] Farhadi A., Harlıoğlu M.M., Gür S. (2019). Artificial extrusion of spermatophores for insemination of the narrow-clawed crayfish *Pontastacus leptodactylus* (Eschscholtz, 1823). Aquac. Rep..

[36] Vuthiphandchai V., Nimrat S., Kotcharat S., Bart A.N. (2007). Development of a cryopreservation protocol for long-term storage of black tiger shrimp (*Penaeus monodon*) spermatophores. Theriogenology.

[37] Farhadi A., Harlıoğlu A.G. (2019). Molecular and cellular biology of the crayfish spermatozoon: Toward development of artificial reproduction in aquaculture. Rev. Fish. Sci. Aquac..

[38] Lezcano M., Granja C., Salazar M. (2004). The use of flow cytometry in the evaluation of cell viability of cryopreserved sperm of the marine shrimp (*Litopenaeus vannamei*). Cryobiology.

[39] Xu X.H., Yan B.L., Xu J.T., Xu G.C., Pu Y.F., Xu J.H. (2010). Cryopreservation of spermatozoa in japanese swimming crab *Charybdis japonica*. Fish. Sci..

[40] Chen D.H. (2006). Cryopreervation of Spermatozoa of *Eriocheir sinensis*. Master’s Thesis.

[41] Liu C.L., Zou Y., Wu Y.Y., Wang Y.J., Liu T., Song A.H., Tang X.X., Liu H.J. (2020). Study on the isolation and cryopreservation of the sperms of *Charybdis japonica*. J. Ocean Univ. Qingdao.

[42] Ma Q., Wang Q., Li K., Ding Y. (2006). Comparative study of trypsinase digestion and homogenization in acquiring free sperms from (Chinese). J. East China Norm. Univ. Shanghai.

[43] Zhang B., Wei P.Y., Xiong J.H., Chen X.H., Zhao Y.Z., Xie D.X. (2014). Preliminary study on semen preparation and sperm quality evaluation of *Ltiopenaeus vannamei*. Southwest China J. Agric. Sci..

[44] Niksirat H., Kouba A., Kozák P. (2014). Post-mating morphological changes in the spermatozoon and spermatophore wall of the crayfish *Astacus leptodactylus*: Insight into a non-motile spermatozoon. Anim. Reprod. Sci..

[45] Wang Q., Misamore M., Jiang C.Q., Browdy C.L. (2010). Egg water induced reaction and biostain assay of sperm from marine shrimp *Penaeus vannamei*: Dietary effects on sperm quality. J. World Aquacult. soc..

[46] Anchordoguy T., Crowe J.H., Griffin F.J., Clark W.H. (1988). Cryopreservation of sperm from the marine shrimp *Sicyonia ingentis*. Cryobiology.

[47] Chen S.L., Liu X.T., Lu D.C., Zhang L.Z., Fang J.P. (1992). Cryopreservation of spermatozoa of silver carp, common carp, blunt snout bream and grass carp. Acta. Zool..

[48] Guan W.B., Wang G.Z., Li S.J., Chen G.F. (2002). Cryopreservation of spermatozoa of mud crab (*Scylla serrata*) and viability assay by biostain method. J. Oceanogr. Taiwan Strait.

[49] Jia P.Y., Guo H.Y., Zhu K.C., Liu B.S., Guo L., Zhang N., Jiang S.G., Zhang D.C. (2021). Cryopreservation of sperm of *Acanthopagrus latus*. South China Fish. Sci..

[50] Hyne R.V. (1984). Bicarbonate- and calcium-dependent induction of rapid guinea pig sperm acrosome reactions by monovalent ionophores. Biol. Reprod..

[51] Ke Y.F., Cai N.E. (1996). Cryopreservation of Spermatozoa from the Marine Shrimp *Penaeus Chinensis*. Oceanol. Limnol. Sin..

[52] Liao X., Ge J.C., Ding S.Y., Chen C.J., Lu Q.P., Huang Y.H. (2008). Study on cryopreservation of the sperm of freshwater prawn *Macrobrachium nipponense* (crustacea, decapoda). J. Nanjing Univ..

[53] Santana J., Cabrita E., Eggen B., Beirão J. (2020). Step by step optimization of a sperm cryopreservation protocol for spotted wolffish (Anarhichas minor Olafsen, 1772). Theriogenology.

[54] Zhou S., Zhu D.F., Wang C.L., Xue L.Y. (2007). Preservation of spermatozoa of blue crab *Portunus trituberculatus*. Mar. Sci..

[55] Arita K., Takamatsu S., Isowa K., Aoki H., Ohta H. (2018). Development of a novel non-programmable cryopreservation method capable of accurate cooling rate manipulation. Aquaculture.

[56] Yang C.L., Zhao Y.Z., Chen X.L., Li Y.M., Peng M., Yang Y.H., He P.P., Chen X.H. (2013). Spermatozoa cryopreservation of *Litopenaeus vannamei*. J. South Agric..

[57] Watson P.F. (2000). The causes of reduced fertility with cryopreserved semen. Anim. Reprod. Sci..

[58] Cartón-García F., Riesco M.F., Cabrita E., Herráez M.P., Robles V. (2013). Quantification of lesions in nuclear and mitochondrial genes of *Sparus aurata* cryopreserved sperm. Aquaculture.

[59] Robles V., Herráez P., Labbé C., Cabrita E., Pšenička M., Valcarce D.G., Riesco M.F. (2017). Molecular basis of spermatogenesis and sperm quality. Gen. Comp. Endocrinol..

[60] Guerriero G., Trocchia S., Abdel-Gawad F.K., Ciarcia G. (2014). Roles of reactive oxygen species in the spermatogenesis regulation. Front. Endocrinol..

[61] Graddis T.J., McMahan C.J., Tamman J., Page K.J., Trager J.B. (2011). Prostatic acid phosphatase expression in human tissues. Int. J. Clin. Exp. Pathol..

[62] Imlay J.A. (2008). Cellular defenses against superoxide and hydrogen peroxide. Annu. Rev. Biochem..

[63] Ren Y., Zhang J., Wang Y., Chen J., Liang C., Li R., Li Q. (2020). Non-specific immune factors differences in coelomic fluid from polian vesicle and coelom of *Apostichopus japonicus*, and their early response after evisceration. Fish Shellfish Immunol..

[64] Dizdaroglu M., Jaruga P., Birincioglu M., Rodriguez H. (2002). Free radical-induced damage to DNA: Mechanisms and measurement. Free Radic. Biol. Med..

[65] Wu D., Cederbaum A.I. (2003). Alcohol, oxidative stress, and free radical damage. Alcohol. Res. Health.

[66] Lopes F., Pinto-Pinho P., Gaivão I., Martins-Bessa A., Gomes Z., Moutinho O., Oliveira M.M., Peixoto F., Pinto-Leite R. (2021). Sperm DNA damage and seminal antioxidant activity in subfertile men. Andrologia.

[67] Afromeev V.I., Tkachenko V.N. (1999). Change in the percent of lactate dehydrogenase isoenzyme level in testes of animals exposed to superhigh frequency radiation. Biofizika.

[68] Liu C., Ren Y.S., Zhang C.X., Yue W.B. (2010). Correlation Analysis of Antioxidant Enzyme Activity and Sperm Motility in Goat Seminal. China Herbiv. Sci..

[69] Yang Z.L., Choi H. (2018). Single-Cell, Time-Lapse Reactive Oxygen Species Detection in *E. coli*. Curr. Protoc. Cell Biol..

[70] Terasaki Y., Terasaki M., Shimizu A. (2021). Protective Effects of Hydrogen against Irradiation. Curr. Pharm. Des..

[71] Liu Z., Yu P., Cai M., Wu D., Zhang M., Chen M., Zhao Y. (2019). Effects of microplastics on the innate immunity and intestinal microflora of juvenile *Eriocheir sinensis*. Sci. Total Environ..

[72] Hong Y., Huang Y., Yan G., Pan C., Zhang J. (2019). Antioxidative status, immunological responses, and heat shock protein expression in hepatopancreas of Chinese mitten crab, *Eriocheir sinensis* under the exposure of glyphosate. Fish Shellfish Immunol..

[73] Xiang Q.M., Wei C.G., Gao X.M., Chen Y.E., Tang D.J., Zhu J.Q., Hou C.C. (2021). Molecular Cloning of Dynein Heavy Chain and the Effect of Dynein Inhibition on the Testicular Function of *Portunus trituberculatus*. Animals.

